# Assessment of protective effect of the losartan against cisplatin-induced nephrotoxicity in mice

**DOI:** 10.1007/s00210-025-04150-7

**Published:** 2025-05-03

**Authors:** Selçuk Teke, Gülsen Bayrak, Erdem Ak, Ali Can Korkmaz, Şakir Necat Yilmaz, Ali Delibaş

**Affiliations:** 1 Department of Pediatrics, Faculty of Medicine, Mersin University, Mersin, Turkey; 2 Department of Histology and Embryology, Faculty of Medicine, Uşak University, Uşak, Turkey; 3 Department of Pediatrics, Pediatric Hematology and Oncology, Faculty of Medicine, Mersin University, Mersin, Turkey; 4 Department of Anatomy, Gulhane Training and Research Hospital, Ankara, Turkey; 5 Department of Histology and Embryology, Faculty of Medicine, Mersin University, Mersin, Turkey; 6 Department of Pediatrics, Pediatric Nephrology, Faculty of Medicine, Mersin University, Mersin, Turkey

**Keywords:** Apoptosis, Proliferation, Losartan, Nephrotoxicity, Cisplatin

## Abstract

Cisplatin is widely used in pediatric oncology but is limited by its dose-dependent nephrotoxicity. The renin–angiotensin–aldosterone system (RAAS) has been implicated in cisplatin-induced renal injury. Losartan, an angiotensin II receptor blocker, may offer renal protection; however, its effects on apoptosis and regeneration in this context remain unclear. This study aimed to investigate the potential protective role of losartan against cisplatin-induced nephrotoxicity, specifically by assessing its impact on apoptosis and tubular regeneration. Fifteen female BALB/c mice were randomly assigned to three groups (*n* = 5 per group): Control, cisplatin (12.7 mg/kg, i.p., single dose), and cisplatin + losartan (10 mg/kg/day, oral). Losartan was administered for nine consecutive days, starting 4 days before cisplatin exposure. Histopathological examination, active caspase-3 immunostaining (for apoptosis), and 5-bromo-2-deoxyuridine (BrdU) labeling (for cell proliferation) were performed. Glomerular and tubular injury scores, caspase-3 H-scores, and BrdU-positive cell counts were statistically analyzed using the Kruskal–Wallis H and Mann–Whitney *U* tests. Cisplatin significantly increased glomerular (*p* = 0.006, *p* = 0.005, *p* = 0.006) and tubular injury scores (*p* = 0.008, *p* = 0.007, *p* = 0.007, *p* = 0.007, *p* = 0.007), elevated active caspase-3 expression (*p* = 0.002), and reduced BrdU-positive cell counts (*p* = 0.009) compared to control. Losartan co-treatment significantly reduced glomerular (*p* = 0.008, *p* = 0.005, *p* = 0.008) and tubular injury (*p* = 0.008, *p* = 0.008, *p* = 0.009, *p* = 0.008, v) and decreased caspase-3 expression (*p* = 0.009). Additionally, BrdU-positive cell counts were significantly higher in the cisplatin + losartan group compared to both control and cisplatin groups (*p* = 0.009), indicating enhanced regeneration. Losartan mitigates cisplatin-induced nephrotoxicity by suppressing apoptosis and promoting tubular regeneration. These findings support the potential therapeutic role of RAAS inhibition in preventing cisplatin-associated renal injury.

## Introductıon

Survival rates in childhood cancers have markedly improved in recent years, largely due to advances in comprehensive and multimodal treatment approaches(Hawkins and Gore 2023). Among these therapeutic agents, cisplatin remains a cornerstone in the management of various pediatric solid tumors, including malignancies of the head and neck, testes, ovaries, as well as osteosarcoma, neuroblastoma, hepatoblastoma, and several types of brain tumors (Ashrafi et al. 2012).

Despite its broad antineoplastic efficacy, cisplatin’s clinical application is significantly constrained by its cumulative, dose-dependent nephrotoxicity (McSweeney et al. 2021). Approximately 30% of patients treated with cisplatin develop AKI. The remaining 70% are at increased risk of long-term chronic kidney disease (Sears and Siskind 2021). The kidneys—particularly the proximal tubular epithelial cells (Williams et al. 2022)—are highly susceptible to cisplatin’s toxic effects, which are mediated through complex mechanisms including deoxyribonucleic acid (DNA) damage, oxidative stress, inflammatory responses, mitochondrial dysfunction, impaired autophagy, apoptosis, and necrosis (McSweeney et al. 2021; Sears and Siskind 2021; Zhang et al. 2023; Abass et al. 2024). Furthermore, cisplatin reduces renal blood flow, thereby aggravating tubular injury and accelerating the onset of AKI (Fang et al. 2021).

In recent years, growing research efforts have focused on the prevention and treatment of cisplatin-induced nephrotoxicity, yielding promising results. These studies have explored several therapeutic strategies, including the use of antioxidants to mitigate oxidative stress, suppression of inflammatory pathways, and pharmacological interventions aimed at reducing cisplatin accumulation within renal cells (Aladaileh et al. 2021; Fang et al. 2021; Zhang et al. 2023).

A major pathophysiological contributor to kidney injury is the overactivation of the renin–angiotensin–aldosterone system (RAAS), which leads to heightened angiotensin II (AT2) activity. AT2 has been shown to promote mesangial cell hypertrophy and proliferation through angiotensin receptors (ATRs) and to enhance superoxide anion production in a dose-dependent manner. Additionally, AT2 elevates glomerular capillary pressure, thereby playing a pivotal role in the development of glomerulosclerosis. Transforming growth factor-beta 1 further facilitates this process by inducing mesangial cell hypertrophy and stimulating extracellular matrix accumulation, both of which are critical drivers of glomerulosclerosis progression (Maires et al. 2022; Arendshorst et al. 2024). Losartan, an angiotensin receptor blocker (ARB), exerts anti-inflammatory effects and mitigates oxidative stress primarily through RAAS inhibition (Nematbakhsh and Ashrafi 2019; Salem et al. 2024; Vajic et al. 2024).

However, the evidence regarding losartan’s protective effect against cisplatin-induced nephrotoxicity remains conflicting. Most existing studies have primarily focused on evaluating serum urea and creatinine levels, along with renal histopathological changes. Yet, the specific impact of losartan on cisplatin-induced cellular injury has not been clearly elucidated (Saleh et al. 2009; Nematbakhsh et al. 2012; Rastghalam et al. 2014; Nematbakhsh and Ashrafi 2019; Tsuji et al. 2022; Salem et al. 2024).

A critical gap in the current literature is the lack of comprehensive investigation into losartan’s effects on apoptotic processes and cellular regeneration within the context of cisplatin nephrotoxicity. Given the complex nature of nephrotoxic injury, simultaneous assessment of apoptosis and regenerative mechanisms is essential for a deeper understanding of the pathophysiology.

The current research is designed to clarify this gap by evaluating losartan’s protective role in cisplatin-induced nephrotoxicity, specifically targeting apoptosis and renal regeneration mechanisms. In our experimental model, apoptotic cells were identified via active caspase- 3 immunohistochemical staining, while DNA replication and cellular regeneration were assessed using anti-BrdU immunohistochemical labeling. This approach provides a novel perspective by evaluating losartan’s protective capacity through both cell death and regenerative pathways, offering insights not previously addressed in the literature.

## Materıals and methods

### Animals

To determine the adequacy of the sample size, a post hoc power analysis was conducted. Based on an effect size of *f* = 18.5532267, the calculated power (1 − β error probability) for the study, which included a total of 15 mice, was 1.0000000. The statistical analysis was conducted using G*Power software (version 3.1.9.7; Franz Faul, University of Kiel, Germany).

The study utilized 15 female BALB/c mice, aged 2 months and weighing between 26 and 33 g. All experimental animals were procured from the University Animal Facility with prior approval obtained from the Institutional Animal Care and Use Committee (Protocol No: 52602694–050/E22273). All procedures were conducted in strict accordance with institutional and ethical guidelines for animal research. The animals were housed in the laboratory under standardized conditions, including a temperature range of 23–25 °C and a 12-h alternating light and dark cycle. Food and water were provided ad libitum. The animals were kept in steel cages and fed with fresh standard feed without any dietary restrictions.

### Experimental animals groups

The mice were selected at random and separated into three groups of five female BALB/c mice each.

- Group 1 (*n* = 5) received saline treatment. (Control group)
- Group 2 (*n* = 5) was treated with cisplatin. (Cisplatin group)
- Group 3 (*n* = 5) was administered losartan and cisplatin. (Cisplatin + Losartan group)

### Experimental design

In experimental animal studies, cisplatin has been shown to induce nephrotoxicity at doses ranging from 6 to 20 mg/kg (Saleh et al. 2009; Ashrafi et al. 2012; Hosoda et al. 2020; Chafik et al. 2022; Tsuji et al. 2022; Airik et al. 2024). Similarly, in previous studies, losartan was administered at a dose of 10–20 mg/kg/day, typically initiated 1 h to 4 days prior to cisplatin exposure and continued for a total duration of 6 to 9 days (Saleh et al. 2009; Haghighi et al. 2012; Rastghalam et al. 2014; Hosoda et al. 2020; Tsuji et al. 2022).

In the present study, cisplatin was administered as a single intraperitoneal (i.p.) dose of 12.7 mg/kg, consistent with the dosing range reported in the literature. Losartan treatment was initiated 4 days prior to cisplatin administration and continued daily. On day 5, a single dose of cisplatin (12.7 mg/kg) was administered concurrently with losartan. Following cisplatin administration, losartan treatment was continued for an additional 4 days, resulting in a total losartan treatment duration of nine consecutive days.

Throughout the experimental phase, the body weight of each animal was monitored daily to ensure accurate dosing.

The control group received an i.p. injection of 5 cc of 0.9% sterile saline. The cisplatin group received a single i.p. dose of cisplatin (Cisplatinteva 10 mg/vial, MEDILAÇ Sanayi ve Ticaret A.Ş., Istanbul, Turkiye) at 12.7 mg/kg. One hour after cisplatin administration, 5 cc of 0.9% sterile saline was administered i.p.

In the cisplatin + losartan group, animals received a single i.p. dose of cisplatin (12.7 mg/kg) following the same protocol. One hour post-cisplatin administration, 5 cc of 0.9% sterile saline was injected i.p. Losartan (Losartan 50 mg, 28 tablets, Drogsan İlaç, Istanbul, Turkiye) was administered orally at a dose of 10 mg/kg/day, starting 4 days prior to cisplatin administration and continuing once daily for a total of 9 days. The final losartan dose was given on the fourth day following cisplatin administration.

To evaluate tubular cell proliferation, 5-bromo- 2-deoxyuridine (BrdU) (BrdU; Sigma, B5002) was administered i.p. at a dose of 200 mg/kg on the fifth day after cisplatin injection.

### Tissue examinations

Four hours after BrdU administration, anesthesia was induced using i.p. injections of ketamine (90 mg/kg) and xylazine hydrochloride (10 mg/kg). Anesthetic adequacy was verified by the lack of reaction to nociceptive stimuli. Following a midline laparotomy, the kidneys were meticulously excised and promptly immersed in freshly prepared 10% neutral buffered formalin for fixation. Following tissue collection, the animals were humanely euthanized by cervical dislocation.

Kidney samples were immersed in 10% neutral buffered formalin and fixed for 48 h. Following fixation, the samples underwent routine processing and were embedded in paraffin. Serial sections, 5 µm thick, were obtained using a rotary microtome (Leica 2125RT) and placed on adhesive-coated slides for subsequent light microscopic and immunohistochemical evaluation.

An experienced histologist, blinded to the group assignments, performed all histological assessments to maintain objectivity and minimize evaluation bias.

#### Histopathological analysis

After deparaffinization, the tissue sections were processed with hematoxylin and eosin (H&E) for histopathological evaluation of the glomeruli and renal tubules. Two sections per animal (*n* = 15) were examined. All evaluations were conducted using a light microscope (BX50; Olympus, Tokyo, Japan), and representative images were captured.

Glomerular damage scoring was performed by modifying a previous study (Demirtaş et al. 2025). A total of 50 glomeruli from ten different randomly selected areas were examined under 200 × magnification for glomerular lobulation, Bowman’s space dilation, and glomerular capillary congestion, and scored as follows: 0, no change; + 1, minimal change; + 2, moderate change; and + 3, severe change.

Tubular damage was indicated at the corticomedullary junction by cell necrosis, tubular dilatation, loss of the brush border, sloughing of cells into the tubular lumen, and formation of tubular casts. These were assessed in ten randomly selected fields per slide at 200 × magnification and scored as follows: 0 = none, 1 = ≤ 10%, 2 = 11–25%, 3 = 26–45%, 4 = 46–75%, and 5 = ≥ 76% (Hazem et al. 2025). Statistical analyses were performed to compare group differences based on these scores.

#### Immunohistochemical analysis

After deparaffinization and rehydration, the sections were transferred to distilled water. For antigen retrieval, sections to be labeled with Anti-BrdU were incubated with trypsin (Abcam, ab970) for 30 min, whereas sections for active caspase- 3 labeling were microwaved in 1X Tris–EDTA (100X, Abcam, ab93684). The sections were then rinsed with phosphate-buffered saline (PBS) and incubated in 3% hydrogen peroxide (H₂O₂) for 10 min to inhibit endogenous peroxidase activity. Sections to be labeled with Anti-BrdU were then incubated in 2 normal hydrochloric acid (HCl) solution at 37 °C for 30 min. To neutralize the HCl effect, the sections were incubated in 0.1 molar di-sodium tetraborate solution for 10 min and washed in tap water. The HCl and di-sodium tetraborate steps were omitted for active caspase- 3 labeling. The sections were rinsed again with phosphate-buffered saline (PBS), circled using a hydrophobic barrier pen, and incubated with normal goat serum (Invitrogen, 50062Z) for 8 min to block nonspecific binding. Subsequently, the sections were incubated overnight at + 4 °C with primary antibodies: Anti-BrdU (1:20, Novocastra) or active caspase- 3 (1:100, Cell Signaling, 9662S). Following primary incubation, Anti-BrdU-labeled sections were treated with an anti-mouse secondary antibody (1:500, Merck Millipore, AP124B), while active caspase- 3-labeled sections were incubated with an anti-rabbit secondary antibody (1:200, Thermo Scientific, 65–6140) for 30 min at room temperature.

Thereafter, horseradish peroxidase (HRP, 1:200, Thermo Scientific, 43–4323) was applied, and the sections were incubated for an additional 10 min at room temperature in the dark. The sections were washed with PBS, developed with the chromogen 3,3′-diaminobenzidine (DAB, Abcam, ab64238), and rinsed in distilled water. Finally, the sections were counterstained with Mayer’s hematoxylin, dehydrated through graded alcohols, and mounted with Entellan.

BrdU-positive cells, identified by dark-brown staining, were quantified in six randomly chosen fields covering the region from the outer medullary stripe to the cortex at 200 × magnification under light microscopy (Dnyanmote et al. 2006).

Cells exhibiting brown perinuclear staining were considered active caspase- 3-positive, and a total of 20 randomly selected areas (ten from the cortex and ten from the outer medulla) were analyzed at 400 × magnification per section (Topcu-Tarladacalisir et al. 2016). Active caspase- 3 expression was evaluated using the histochemical score (H-Score) method, which integrates staining intensity (i) and the percentage of positively stained cells at each intensity level (Pi). Staining intensity was graded as 0 (no staining), 1 (weak), 2 (moderate), and 3 (strong). The Pi values ranged from 0 to 100%. The H-Score was calculated using the formula: H-Score = (0 × P0) + (1 × P1) + (2 × P2) + (3 × P3), yielding a total score between 0 and 300 (Pranoto et al. 2024).

Microscopic assessments were performed with a light microscope (BX50; Olympus, Tokyo, Japan), and photomicrographs were obtained. Statistical analyses were performed to compare group differences based on these scores.

### Statistical considerations

Statistical analyses were performed using SPSS version 22 (IBM Corp., Armonk, NY, USA). Given that each experimental group comprised five animals, the Kruskal–Wallis H test was used to evaluate overall differences in pathological findings across the three groups for all staining assessments. For parameters showing significant differences, pairwise comparisons between groups were conducted using the Mann–Whitney *U* test. A *p* value of less than 0.05 was considered statistically significant.

## Results

### Histopathological findings

**Fig. 1:**
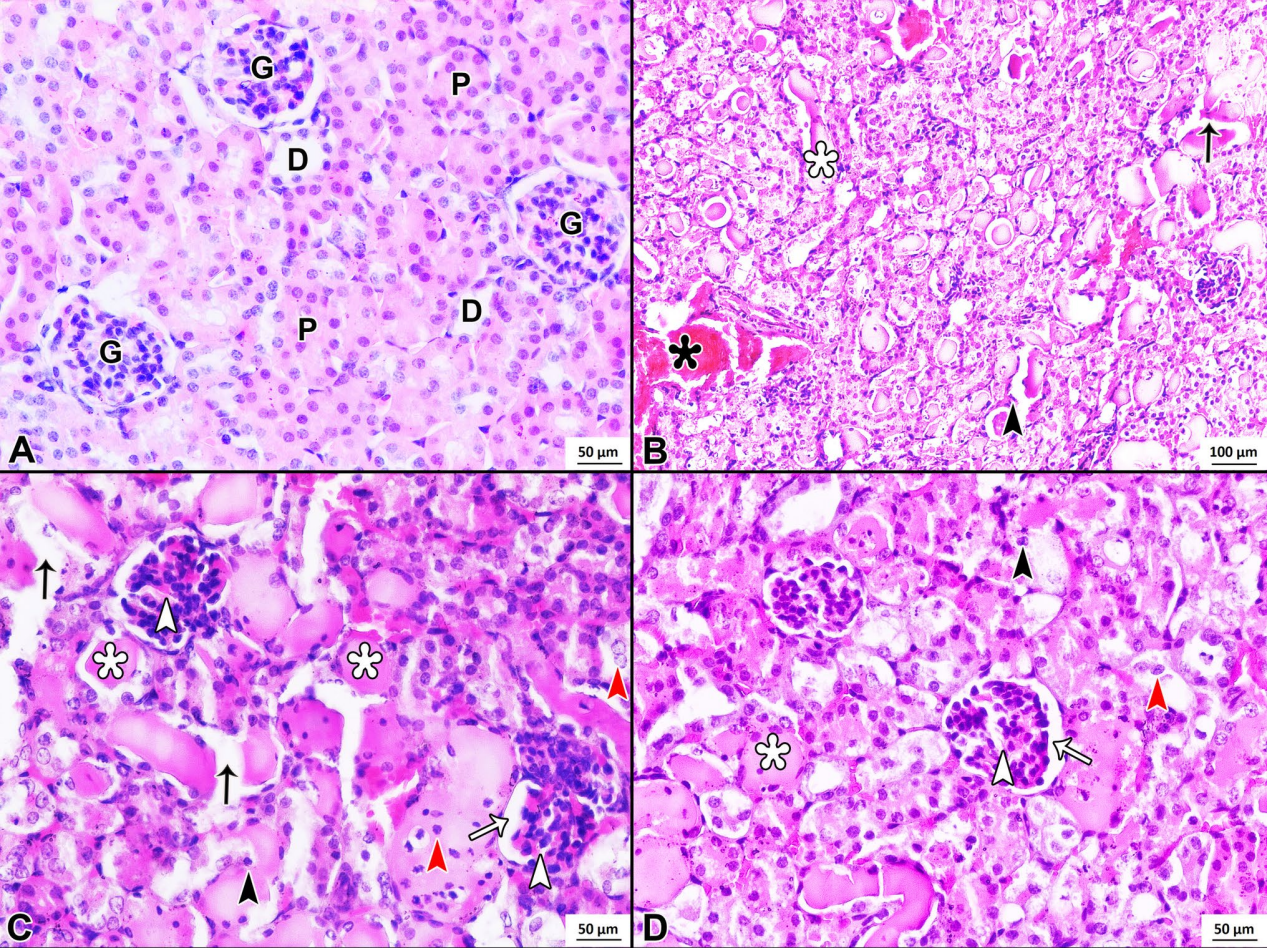
H&E staining of control (**A**), cisplatin (**B** and **C**), and cisplatin + losartan (**D**) groups. In the control group, glomeruli (G), proximal (P), and distal (D) tubules with normal morphological features. In the cisplatin group, glomerular lobulation and glomerular capillary congestion (white arrowhead), Bowman’s space dilation (white arrow), loss of the brush border and necrotic cells with pyknotic nuclei (black arrowhead), sloughing tubular epithelial cells into the lumen (red arrowhead), tubular dilation and degeneration (black arrow), tubular cast formation (white asterisk), and congestion in the blood vessels (black asterisk). In the cisplatin + losartan group, loss of brush border and necrotic cell with pyknotic nuclei (black arrowhead), sloughing cells into the lumen (red arrowhead), and tubular cast formation (white asterisk) in some tubules. Glomerular lobulation and glomerular capillary congestion (white arrowhead), Bowman’s space dilation (white arrow). Magnification: A, C, and D 200 ×; B 40 ×

H&E stained sections from the control group (Fig. 1A) exhibited normal glomeruli and both normal proximal and distal (D) tubules with typical morphological features. The glomerular capillaries and Bowman’s space appeared normal. The tubular epithelial cells and brush border exhibited normal histology.

When examining the H&E sections of the cisplatin group (Fig. 1B, C), lobulation was observed in the glomeruli. Glomerular capillaries showed congestion, and Bowman’s space was expanded. Some glomeruli had atrophic features. Tubular damage was quite pronounced in this group. A marked loss of the brush border was observed in the tubular epithelial cells, which exhibited necrotic features along with pyknotic nuclei. In many tubules, epithelial cells had sloughed into the lumen. Tubular cast formation, tubular dilation, and tubular degeneration were observed in many tubules. Besides, congestion was observed in some of the blood vessels. Minimal infiltration of inflammatory cells was found in certain areas.

In the cisplatin + losartan group, H&E sections (Fig. 1D) showed lobulation in some glomeruli, glomerular capillary congestion, and widening of Bowman’s space. Tubular degeneration was reduced compared to the cisplatin group, but some tubular epithelial cells had sloughed into the lumen. Cast formation was observed in some tubules.

Statistical analysis was conducted using the Kruskal–Wallis H test to compare pathological findings among the three groups, as summarized in Table 1. The analysis revealed a statistically significant difference among the groups (*p* < 0.01).

**Table 1:** A comparison of pathological findings examined by hematoxylin and eosin staining

Pathological findings	Group 1 (Control)	Group 2 (Cisplatin)	Group 3 (Cisplatin + Losartan)	*p*
	Mean ± SD	Mean ± SD	Mean ± SD	
Cell necrosis	0.16 ± 0.11	4.1 ± 0.17	2.92 ± 0.23	0.002*
Tubular dilatation	0.14 ± 0.05	4.42 ± 0.27	2.94 ± 0.18	0.002*
Loss off the brush border	0.12 ± 0.04	4.02 ± 0.31	3.2 ± 0.12	0.002*
Sloughing of cells into the tubular lumen	0.14 ± 0.19	4.06 ± 0.15	2.92 ± 0.13	0.002*
Formation of tubular cast	0.02 ± 0.04	4.22 ± 0.19	2.78 ± 0.24	0.002*
Glomerular lobulation	0.12 ± 0.04	2.9 ± 0.07	1.96 ± 0.21	0.002*
Bowman’s space dilation	0.14 ± 0.05	2.8 ± 0.00	1.92 ± 0.22	0.001*
Glomerular capillary congestion	0.12 ± 0.04	2.84 ± 0.05	1.94 ± 0.27	0.002*

*p*: Kruskal Wallis H Test *p* value *p < 0.01

**Fig. 2:**
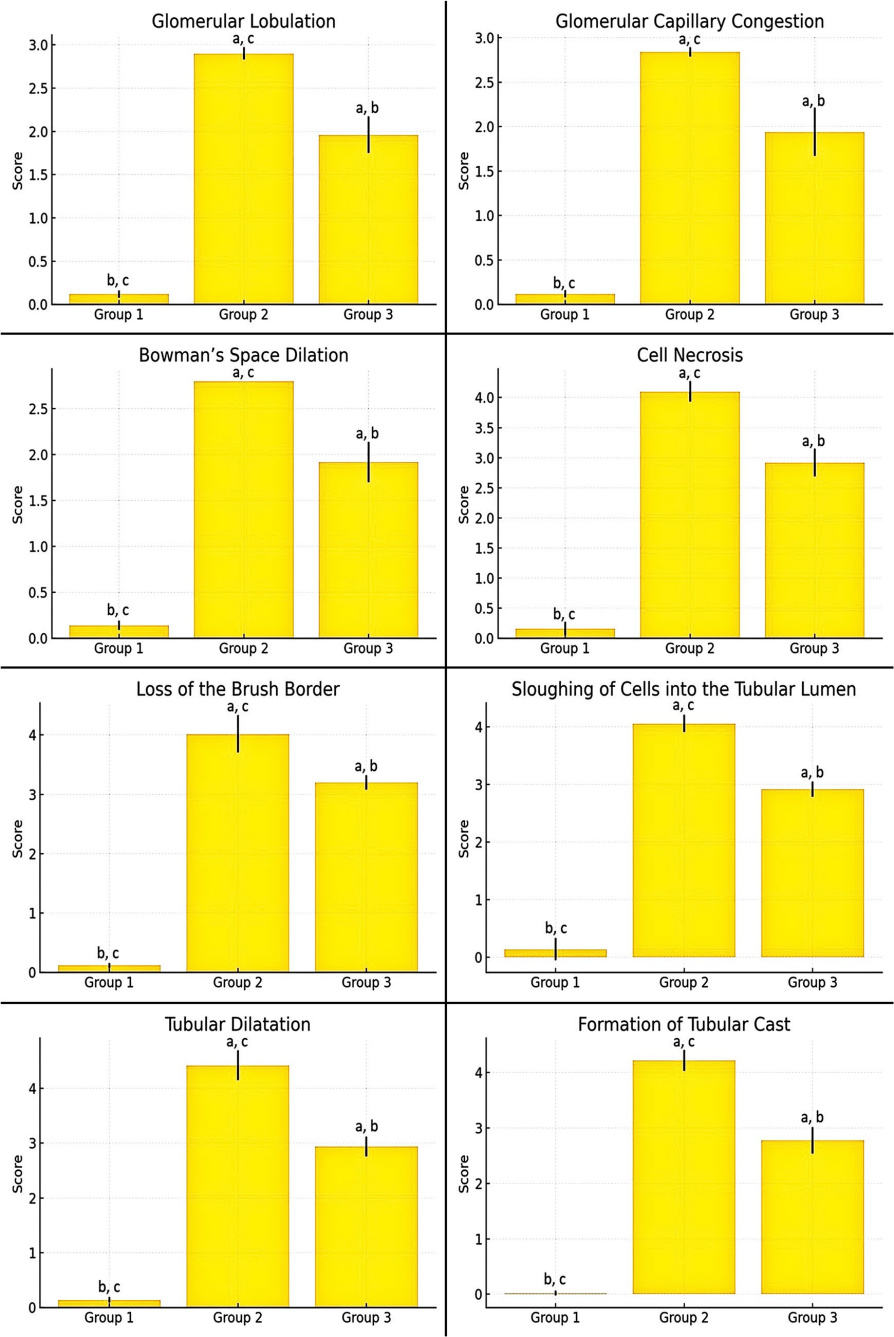
Comparison of glomerular and tubular damage between groups. ^a^The difference between this group and control group was statistically significant. ^b^The difference between this group and cisplatin group was statistically significant. ^c^The difference between this group and cisplatin + losartan group was statistically significant

To further identify the source of this difference, pairwise comparisons were conducted using the Mann–Whitney *U* test, a non-parametric method suitable for independent two-sample comparisons. The detailed results of these analyses are presented in Table 2, illustrating the pathological findings between groups. Regarding the scoring of glomerular damage, glomerular lobulation, glomerular capillary congestion, and Bowman’s space dilation were statistically significantly increased in the cisplatin group relative to the control group, while in the cisplatin + losartan group, these parameters showed a marked decrease in comparison with the cisplatin group (Fig. 2, Tables 1 and 2).

**Table 2:** Comparison of pathological findings among experimental groups using hematoxylin and eosin staining

Pathological findings	Group 1–2		Group 1–3		Group 2–3	
	*p*	*z*	*p*	*z*	*p*	*z*
Cell necrosis	0.008*	− 2.652	0.009*	− 2.627	0.008*	− 2.652
Tubular dilatation	0.007*	− 2.685	0.008*	− 2.660	0.008*	− 2.652
Loss off the brush border	0.007*	− 2.694	0.007*	− 2.712	0.009*	− 2.627
Sloughing of cells into the tubular lumen	0.007*	− 2.703	0.006*	− 2.730	0.008*	− 2.652
Formation of tubular cast	0.007*	− 2.694	0.006*	− 2.730	0.008*	− 2.643
Glomerular lobulation	0.006*	− 2.730	0.007*	− 2.694	0.008*	− 2.643
Bowman’s space dilation	0.005*	− 2.835	0.008*	− 2.660	0.005*	− 2.795
Glomerular capillary congestion	0.006*	− 2.739	0.007*	− 2.694	0.008*	− 2.652

*p*: Mann–Whitney *U* test *p* value *p < 0.05

For tubular damage scoring, cell necrosis, tubular dilation, tubular cast formation, sloughed epithelial cells into the lumen, and loss of brush border were statistically increased in the cisplatin group compared to the control group. In the cisplatin + losartan group, tubular damage scores were statistically reduced compared to the cisplatin group (Fig. 2, Tables 1 and 2).

### Immunohistochemical findings

#### Evaluation of active caspase- 3 labeling

**Fig. 3:**
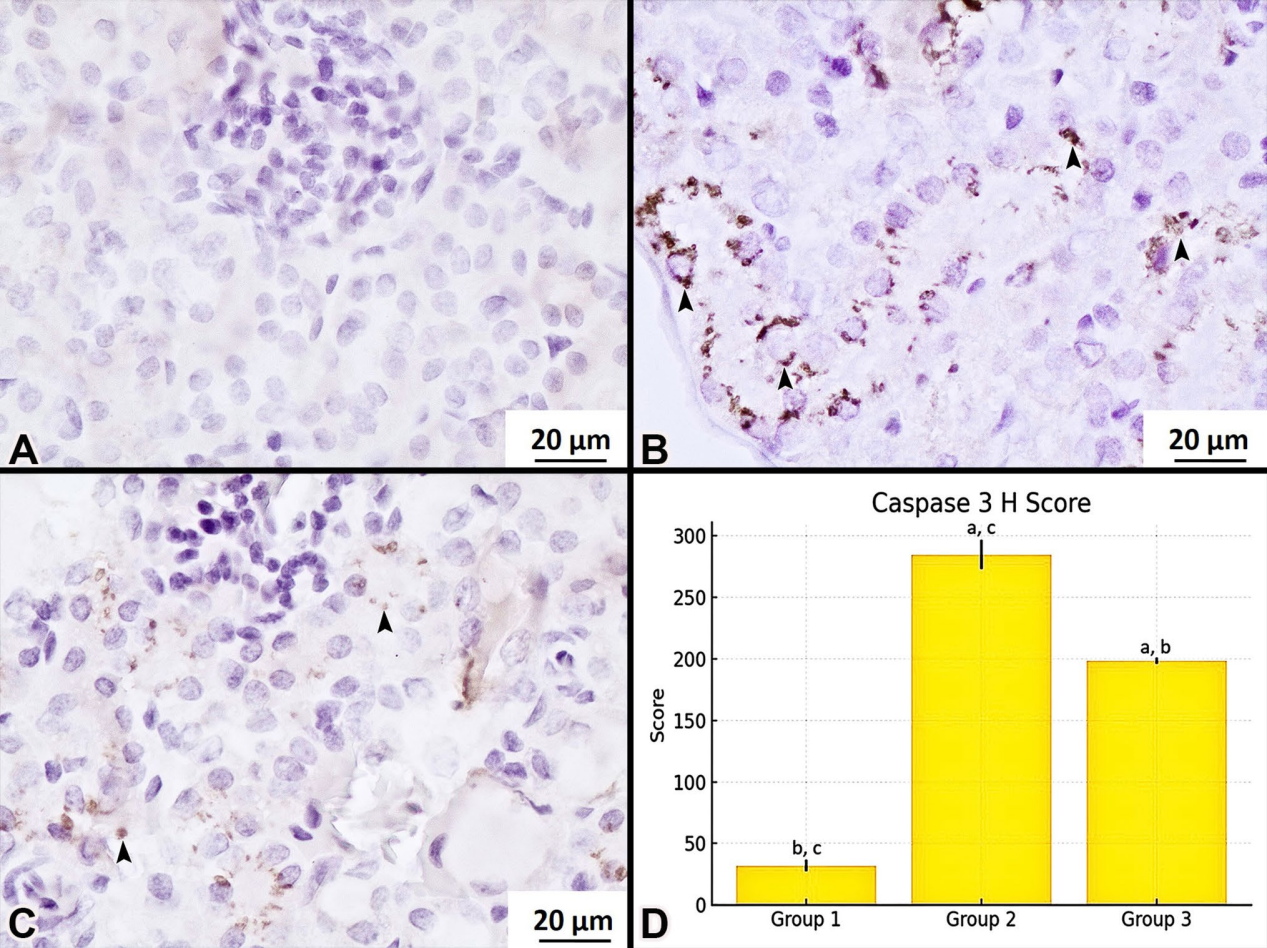
Caspase- 3 immunostaining. Control (**A**), cisplatin (**B**), cisplatin + losartan (**C**). Positive immunostaining (black arrowhead). Comparison of H score (**D**) between groups. ^a^The difference between this group and control group was statistically significant. ^b^The difference between this group and cisplatin group was statistically significant. ^c^The difference between this group and cisplatin + losartan group was statistically significant. Magnification 400 ×

Active caspase- 3 immunostaining revealed minimal or absent staining in the control group, with only occasional areas showing faint positivity (Fig. 3A). In contrast, the cisplatin group exhibited strong positive staining for active caspase- 3, indicating marked apoptotic activity (Fig. 3B). Notably, the cisplatin + losartan group demonstrated moderate staining intensity, suggesting a reduction in apoptosis compared to the cisplatin group (Fig. 3C).

The H-scores of active caspase- 3 immunostaining were compared across groups using the Kruskal–Wallis H test, which revealed a statistically significant difference (*p* = 0.002) (Table 3). Subsequently, the Mann–Whitney *U* test was applied for pairwise comparisons between groups to further evaluate significant differences. The results of these analyses are presented in Table 4.

**Table 3:** Comparison of pathological findings among groups based on active caspase- 3 expression

Pathological findings	Group 1 (Control)	Group 2 (Cisplatin)	Group 3 (Cisplatin + Losartan)	*p*
	Mean ± SD	Mean ± SD	Mean ± SD	
Caspase- 3 H score	32.08 ± 4.56	284.77 ± 11.73	198.7 ± 2.41	0.002*

*p*: Kruskal Wallis H Test *p* value *p < 0.01

**Table 4:** Comparison of pathological findings among experimental groups using active caspase- 3 staining

Pathological findings	Group 1–Group 2		Group 1–Group 3		Group 2–Group 3	
	*p*	*z*	*p*	*z*	*p*	*z*
Caspase 3 H score	0.009*	− 2.619	0.009*	− 2.619	0.009*	− 2.611

*p*: Mann–Whitney *U* test *p* value *p < 0.05

H-score analysis revealed that active caspase- 3 positive staining was significantly higher in the cisplatin group compared to the control group (*p* = 0.009) (Fig. 3D, Tables 3 and 4). Conversely, the cisplatin + losartan group demonstrated a statistically significant reduction in positive staining compared to the cisplatin group (*p* = 0.009) (Fig. 3D, Tables 3 and 4).

#### Evaluation of anti-BrdU labeling

**Fig. 4:**
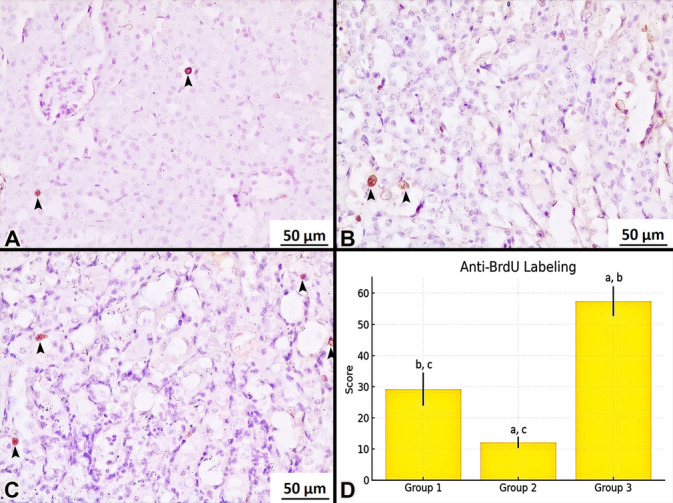
BrdU immunostaining. Control (**A**), cisplatin (**B**), cisplatin + losartan (**C**). Positive immunostaining (black arrowhead). Comparison of positive staining cell (**D**) between groups. ^a^The difference between this group and control group was statistically significant. ^b^The difference between this group and cisplatin group was statistically significant. ^c^The difference between this group and cisplatin + losartan group was statistically significant. Magnification 200 ×

Examination of anti-BrdU-labeled sections revealed positively stained cells in the control group (Fig. 4A). In contrast, the cisplatin group exhibited a significant decrease in BrdU-positive cell counts (Fig. 4B). Notably, the cisplatin + losartan group demonstrated a significant increase in BrdU-positive cells compared to both the control and cisplatin groups (Fig. 4C).

Following the evaluation of anti-BrdU-labeled sections, the mean and standard deviation of positively stained cell counts were calculated for each group. The Kruskal–Wallis H test was performed to assess overall differences among the three groups, with the results summarized in Table 5. This analysis revealed a statistically significant difference (*p* < 0.01).

**Table 5:** Comparison of anti-BrdU cell numbers according to experimental groups

	Anti-BrdU	
	Mean ± SD	*p*
Group 1 (Control)	29.20 ± 5.29	0.002*
Group 2 (Cisplatin)	12.10 ± 1.82	
Group 3 (Cisplatin + Losartan)	57.40 ± 4.72	

*p*: Kruskal Wallis H Test *p* value *p < 0.01

To further explore the source of this variation, pairwise comparisons were conducted using the Mann–Whitney *U* test. The detailed results of these comparisons are presented in Table 6, providing a comprehensive comparison of cell counts between groups.

**Table 6:** Comparison of anti-BrdU cell numbers between experimental groups

	Anti-BrdU	
	*p*	*z*
Group 1–Group 2	0.009*	− 2.619
Group 1–Group 3	0.009*	− 2.611
Group 2–Group 3	0.009*	− 2.619

*p*: Mann–Whitney *U* test *p* value *p < 0.05

Anti-BrdU cell count analysis demonstrated a significant reduction in the number of positively stained cells in the cisplatin group compared to the control group (*p* = 0.009) (Fig. 4D, Tables 5 and 6). In contrast, the cisplatin + losartan group exhibited a statistically significant increase in BrdU-positive cell counts compared to both the control and cisplatin groups (*p* = 0.009) (Fig. 4D, Tables 5 and 6).

#### Effect size analysis

In addition to statistical significance, effect size values (*η*^2^) were calculated to evaluate the magnitude of differences among the three study groups (*n* = 15). The Kruskal–Wallis H test was performed using SPSS, and chi-squared (H) and significance (*p*) values were obtained for each parameter. Effect sizes were then calculated using the formula *η*^2^ = (*H* − *k* + 1)/(*n* − *k*), where *H* represents the test statistic, *k* is the number of groups, and *n* is the total sample size. As presented in Table 7, these values reflect the strength of the group-level differences for each pathological and immunohistochemical parameter. All evaluated parameters demonstrated large effect sizes, indicating strong and meaningful differences among the study groups.

**Table 7:** Effect size (*η*^2^) values based on Kruskal–Wallis H statistics for histopathological and immunohistochemical parameters

Parameters	Chi-square	Eta squared (*η*^2^)
Cell necrosis	12.635	0.886
Tubular dilatation	12.727	0.894
Loss off the brush border	12.774	0.898
Sloughing of cells into the tubular lumen	12.844	0.904
Formation of tubular cast	12.821	0.902
Glomerular lobulation	12.821	0.902
Bowman’s space dilation	13.109	0.926
Glomerular capillary congestion	12.844	0.904
Caspase- 3 h score	12.522	0.877
Anti-brdu	12.522	0.877

## Discussion

Our findings revealed marked glomerular alterations, such as glomerular lobulation, Bowman’s space dilation, glomerular capillary congestion, alongside pronounced tubular damage characterized by cell necrosis, tubular dilation, brush border loss, luminal cell sloughing, and cast formation following cisplatin exposure. Additionally, an increase in apoptosis, evidenced by active caspase- 3 staining, and a decrease in regenerative capacity, demonstrated by reduced BrdU-positive cell counts, were detected. These findings align with existing literature highlighting the central role of apoptosis in cisplatin-induced nephrotoxicity (Topcu-Tarladacalisir et al. 2016; Airik et al. 2024). Similarly, Airik et al. (2024) reported the induction of oxidative stress, apoptosis, and ferroptosis in a murine model treated with a single dose of 20 mg/kg cisplatin.

The potential renoprotective effect of the angiotensin receptor blocker (ARB) losartan against cisplatin-induced kidney injury has been investigated in several studies, yielding conflicting results (Saleh et al. 2009; Ashrafi et al. 2012; Rastghalam et al. 2014; Nematbakhsh et al. 2017; Nematbakhsh and Ashrafi 2019; Salem et al. 2024). While some studies reported reductions in serum creatinine levels and histological damage, others found no protective effect or even exacerbation of nephrotoxicity. Such discrepancies are likely due to methodological factors, including variations in cisplatin dose, treatment protocols, animal models, and sex differences.

In our study, losartan treatment markedly attenuated both glomerular and tubular injury, suppressed apoptosis, and enhanced regenerative capacity. These findings suggest that losartan confers nephroprotection by modulating pathways involved in cellular injury and repair. At the molecular level, excessive activation of the RAAS leads to elevated levels of ATII, which promotes oxidative stress, inflammation, mesangial cell hypertrophy, and ultimately glomerulosclerosis (Maires et al. 2022; Arendshorst et al. 2024). By antagonizing the profibrotic, proinflammatory, and vasoconstrictive actions of angiotensin II, losartan downregulates TGF-β gene expression and mitigates the risk of glomerulosclerosis (Rajabi et al. 2025). Additionally, experimental studies have demonstrated that losartan preserves renal architecture by attenuating oxidative stress and inflammation, as well as reducing lipid peroxidation and protein carbonylation (Şahin et al. 2024; Vajic et al. 2024). Furthermore, losartan inhibits key pathological mechanisms triggered by RAAS activation, including mitochondrial dysfunction, impaired autophagy, and upregulation of SGLT2 expression, thereby preventing both mitochondrial injury and hypertensive renal damage (Miyata et al. 2021; Yin et al. 2024). Collectively, these findings—consistent with our results—underscore losartan’s nephroprotective potential through its multifaceted modulation of injury and repair pathways.

Despite employing a relatively high cisplatin dose of 12.7 mg/kg, which exceeds that used in many prior studies, the administration of losartan at 10 mg/kg/day for nine consecutive days provided significant nephroprotection. This finding highlights the crucial role of treatment duration and protocol design in determining the protective efficacy. For instance, Hosoda et al. (2020) reported that single-dose losartan worsened nephrotoxicity, while Nematbakhsh et al. (2012) observed no significant protection with only 4 days of losartan treatment. In contrast, our extended and consistent treatment regimen effectively revealed losartan’s anti-apoptotic and regenerative properties.

Furthermore, Rastghalam et al. (2014) reported that losartan at 10 mg/kg was ineffective, and a higher dose of 20 mg/kg even aggravated nephrotoxicity. Our findings confirm that losartan, when administered at an appropriate dose and duration, significantly mitigates cisplatin-induced renal injury by suppressing apoptosis. This highlights the dose-dependent nature of losartan’s protective effect and suggests that excessive dosing may be detrimental.

Additionally, Tsuji et al. (2022) demonstrated that while telmisartan and enalapril exacerbated cisplatin nephrotoxicity, losartan did not exhibit similar adverse effects. Losartan, unlike other RAS inhibitors, maintained comparable renal function tests and histological findings to the control group. This distinction is likely due to losartan’s selective AT1 receptor antagonism and lack of PPAR-γ agonist activity. These results support the safe use of losartan during cisplatin therapy without exacerbating renal injury.

Notably, our study utilized only female mice and nevertheless demonstrated a significant nephroprotective effect of losartan. Haghighi et al. (2012) and Nematbakhsh et al. (2012) previously reported sex-dependent effects, with losartan being protective in male rats but exacerbating nephrotoxicity in females. Interestingly, this adverse effect was absent in ovariectomized females, suggesting a modulatory role of sex hormones, particularly estrogen, on RAAS interactions. The differences in our findings may stem from species variations (mice vs. rats), oral administration of losartan, and the extended treatment duration. Our results indicate that, with appropriate dosing and administration protocols, losartan can also exert protective effects in female models.

Additionally, Salem et al. (2024) demonstrated that encapsulating losartan within nanocubic vesicles mitigated acute kidney injury through apoptosis suppression and activation of the Wnt/β-catenin/TCF- 4 pathway. Their approach emphasizes targeted delivery to renal tissue, potentially minimizing systemic side effects. While our study achieved similar protective outcomes through conventional administration, future integration of nanotechnological carriers may further enhance losartan’s therapeutic efficacy and safety.

Our study possesses several notable strengths. Despite reports suggesting limited protective effects of losartan in female animal models, our findings clearly demonstrate significant nephroprotection in female mice. This supports the notion that losartan’s protective potential may be independent of sex, provided optimal dosing and administration protocols are employed.

Additionally, our study extended beyond conventional histopathological assessments by incorporating active caspase- 3 and BrdU immunohistochemical analyses, directly evaluating apoptosis and regenerative processes. This robust methodological approach objectively demonstrated losartan’s dual protective and reparative effects at the cellular level.

Finally, our 9-day treatment protocol, encompassing both pre- and post-cisplatin administration, represents a major advantage over shorter regimens reported in the literature. This design likely contributed to the clear demonstration of losartan’s protective effects.

Due to ethical considerations, the sample size was limited to 15 female BALB/c mice, which may have impacted the statistical power and generalizability of the results. Although a larger sample size could have further strengthened the experimental validity, the study was designed in compliance with the “minimum animal use” principle in accordance with ethical guidelines for animal research. Furthermore, all experiments were conducted exclusively in female mice. Given that previous studies have reported sex-dependent responses to losartan, future research including both sexes would be valuable (Haghighi et al. 2012; Nematbakhsh et al. 2012). Finally, we were unable to perform standard biochemical assessments of renal function, such as serum creatinine and BUN measurements. Although our histopathological and immunohistochemical analyses provide robust data, the absence of functional evaluations limits the comprehensive assessment of renal impairment.

Our study provides compelling evidence that losartan exerts a strong nephroprotective effect against cisplatin-induced renal injury by reducing apoptosis and enhancing regenerative capacity. These findings support RAAS modulation as a promising therapeutic target for mitigating cisplatin nephrotoxicity. Moreover, they highlight the critical roles of dosage, treatment duration, and sex as key factors influencing therapeutic efficacy. Future studies exploring losartan in combination with nanotechnological delivery systems may offer more effective and targeted renal protective strategies.

## Data Availability

All source data for this work (or generated in this study) are available upon reasonable request.
